# NURSE STAFFING CHARACTERISTICS AT US NURSING HOMES: NURSING MIX AND INTENSITY

**DOI:** 10.1093/geroni/igad104.0748

**Published:** 2023-12-21

**Authors:** Gregory Orewa, Ganisher Davlyatov, Akbar Ghiasi, Robert Weech-Maldonado

**Affiliations:** University of Texas, San Antonio, San Antonio, Texas, United States; University of Oklahoma Health Sciences Center, Oklahoma City, Oklahoma, United States; University of the Incarnate Word, San Antonio, Texas, United States; University of Alabama at Birmingham, Birmingham, Alabama, United States

## Abstract

NHs have long struggled with lack of appropriate and consistent supply of nursing staff. Recruitment of qualified nursing staff is difficult due to the relatively low pay, supply-demand mismatch, and onerous regulatory and administrative demands. Research suggests that approximately 80% of NHs are understaffed. Despite NHs offering financial incentives to attract staff nurses, the use of contract nurses has been on the rise with 78% facilities reporting using agency nurse staff. However, much of this evidence remains anecdotal or precedes the COVID 19 pandemic, which had a disproportionate impact on the NH industry. The purpose of this research was to explore the overall nursing staffing trends at US NHs from 2017-2022. The study used the CMS Payroll Based Journal (PBJ) data for years 2017-2022. We examined the nursing staffing intensity, the proportion of contract nurses to total nurses, and the proportion of registered nurses (RN) to total nurse staffing. Our results suggest that the RN and licensed practical nurses (LPN) hours per resident day (PRD) stayed relatively consistent through 2017-2022, with a slight increase noted during the COVID-19 pandemic. There was a significant decrease in CNA hours PRD in 2022 compared to 2017. Contract nurse utilization across RNs, LPNs, and CNAs increased in 2022 compared to 2017. Looking at the trends, contract nurse utilization has been consistently increasing, but it may have peaked during the pandemic. RN to total nurse ratio increased in 2022 compared to 2017, though noticeably, the sharpest increase was during the pandemic.

